# Supplementary material for: Myelination across cortical hierarchies and depths in humans and macaques
Source: bioRxiv. 2025 Feb 6:2025.02.06.636851. Preprint. [Version 1] doi: 10.1101/2025.02.06.636851 (PMC11839058; doi:10.1101/2025.02.06.636851)
Supplement: Supplement 1 [file NIHPP2025.02.06.636851v1-supplement-1.pdf]

bioRxiv preprint doi: https://doi.org/10.1101/2025.02.06.636851; this version posted February 6, 2025. The copyright holder for this preprint (which was not certified by peer review) is the author/funder, who has granted bioRxiv a license to display the preprint in perpetuity. It is made available under aCC-BY 4.0 International license.

Supplementary Results & Figures

Validation of cortical myelin imaging with MBP expression in macaques

Although imaging modalities such as T1w/T2w ratio, magnetization transfer (MT), and myelin water fraction (MWF) can indirectly reflect myelin content across the cortical surface[24–26,32,42–44,77,78], this relationship is imperfect[79,80], and their specificity across cortical depths remains less established[18,81]. To assess the correlation between the T1w/T2w ratio and myelin content across both regions and cortical depth[10,39], we compared it with the expression of myelin basic protein (MBP) in macaques using single-cell sequence[45]. Spatially, MBP expression was higher in sensorimotor regions than in association regions (Fig. S2A, B R^2 = 0.250, $F(1, 131)$ = 24.350, $P < 0.001$). The T1w/T2w ratio showed a significant positive correlation with MBP expression across cortex (Fig. S2C R^2 = 0.216, F(1, 131) = 37.3, $P < 0.001$), consistent with prior findings [24,42–44,77,78,82].

For depth-wise validation, MBP expression was also significantly higher in deeper layers than in superficial layers (Fig. S2D, E, all ANOVAs $F > 9.236$, $P < 0.001$). To assess finer grained correspondence within individual cortical areas, we calculated the correlation coefficient between MBP and the T1w/T2w ratio for each CHARM parcel (Fig. S2F). Among the 86 parcels that had six histological layers identified in the MBP data, 88% (76 parcels) showed a significant correlation ($r > 0.815$, $P < 0.050$). A region-wise permutation test confirmed that this depth-dependent correlation is significantly higher than expected by random chance ($T = -108.309$, $P < 0.001$). These consistent depth-dependent patterns across broad cortical regions and the high proportion of significant correlations at the individual areas level validate the T1w/T2w ratio as a reliable marker for myelin content across both species and provide a robust method for assessing myelination variations across regions and depths.

bioRxiv preprint doi: https://doi.org/10.1101/2025.02.06.636851; this version posted February 6, 2025. The copyright holder for this preprint (which was not certified by peer review) is the author/funder, who has granted bioRxiv a license to display the preprint in perpetuity. It is made available under aCC-BY 4.0 International license.

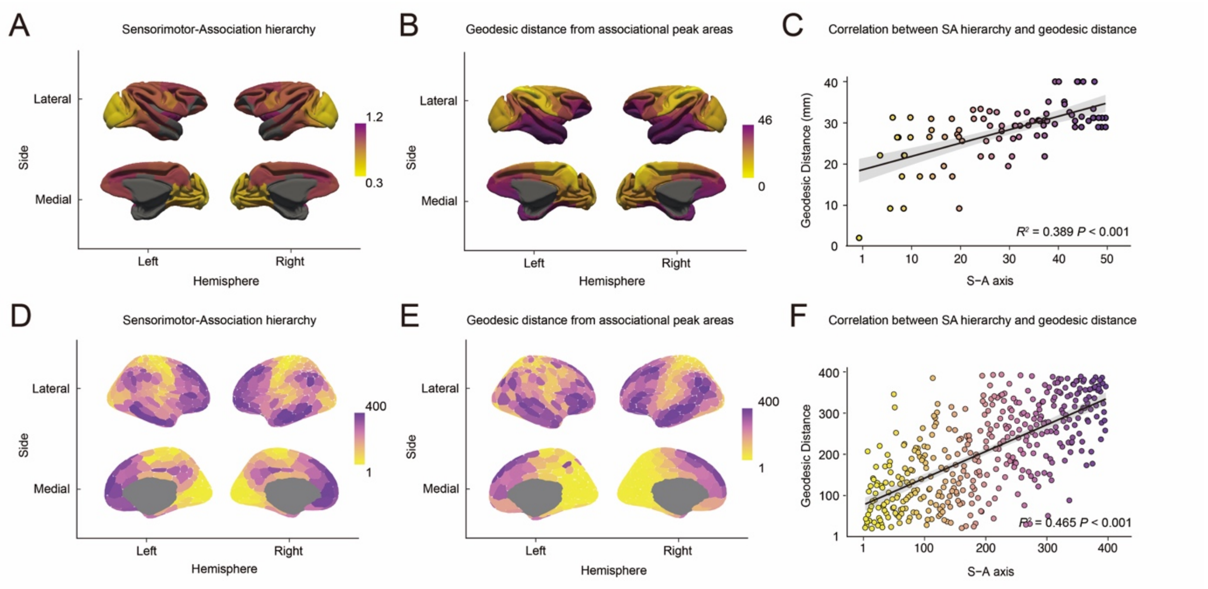

Supplementary Figure 1. Correlation between geodesic distance and the sensorimotor-association axis. (A, D) Sensorimotor-association axis in macaques (A) and humans (D). **(B, E)** Geodesic distance from association regions in macaques (B) and humans (E). **(C, F)** Correlation between S-A axis and geodesic distance in macaques (C, $R^2 = 0.389$, $P < 0.001$) and humans (F, $R^2 = 0.465$, $P < 0.001$).

bioRxiv preprint doi: https://doi.org/10.1101/2025.02.06.636851; this version posted February 6, 2025. The copyright holder for this preprint (which was not certified by peer review) is the author/funder, who has granted bioRxiv a license to display the preprint in perpetuity. It is made available under aCC-BY 4.0 International license.

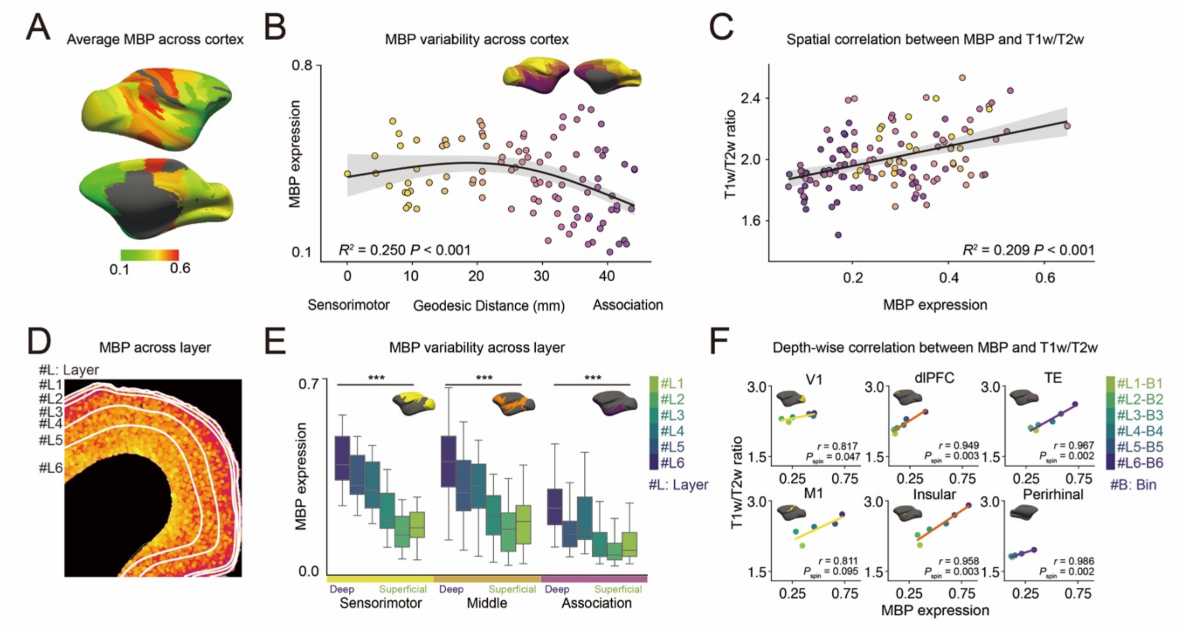

Supplementary Figure 2. Correlation between MBP and T1w/T2w ratio across layers in macaques. (A) MBP expression across the cortex. **(B)** MBP expression along the geodesic distance from association regions. Brain regions are arranged on a spectrum from farther (yellow) to closer (purple) to the association centers. The GAM-predicted fit is displayed with a 95% confidence interval (R^2 = 0.250, P < 0.001). **(C)** Correlation between MBP expression and the T1w/T2w ratio across the cortex (R^2 = 0.209, P < 0.001). The regression line is displayed with a 95% confidence interval (R^2 = 0.209, P < 0.001). **(D)** Schematic illustration of MBP expression across cortical layers. **(E)** MBP expression in each layer for sensorimotor (yellow), middle (orange), and association (purple) regions. ANOVA $^{***}P$ < 0.001. **(F)** Correlation between MBP expression and T1w/T2w ratio across layers or bins in representative brain regions.

bioRxiv preprint doi: https://doi.org/10.1101/2025.02.06.636851; this version posted February 6, 2025. The copyright holder for this preprint (which was not certified by peer review) is the author/funder, who has granted bioRxiv a license to display the preprint in perpetuity. It is made available under aCC-BY 4.0 International license.

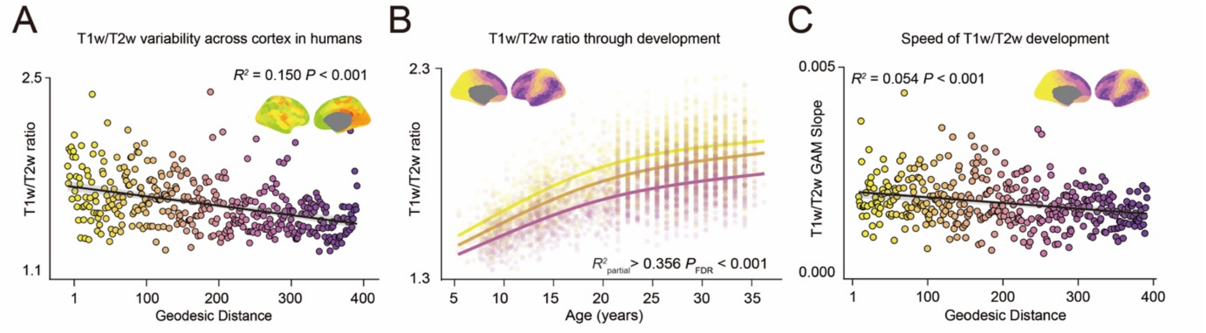

Supplementary Figure 3. Development of T1w/T2w ratio along geodesic distance in humans. **(A)** T1w/T2w ratio across the cortex in humans. Brain parcels are aligned along geodesic distance from default mode network regions ($R^2 = 0.150$, $P < 0.001$). (B) Developmental trajectories of the T1w/T2w ratio for sensorimotor (yellow), middle (orange) and association (purple) cortical areas. Solid lines illustrate Generalized Additive Model (GAM)-predicted fits along with their 95% confidence intervals (Sensorimotor; $R^2_{partial} = 0.466$, $P_{FDR} < 0.001$, Middle; $R^2_{partial} = 0.430$, $P_{FDR} < 0.001$, Association; $R^2_{partial} = 0.356$, $P_{FDR} < 0.001$). **(C)** GAM slope along the geodesic distance for humans. The GAM-predicted fit is displayed with a 95% confidence interval ($R^2 = 0.054$, $P < 0.001$).
